# Supplementary material for: Robotic middle ear access for cochlear implantation: First in man
Source: PLoS One. 2019 Aug 2;14(8):e0220543. doi: 10.1371/journal.pone.0220543 (PMC6677292; doi:10.1371/journal.pone.0220543)
Supplement: S1 File — (PDF) [file pone.0220543.s001.pdf]

Studienprotokoll

Machbarkeitsstudie

Roboterassistierte Cochleaimplantation

Version 1.0 vom 10. September 2013

Antragsteller

Universitätsklinik für Hals-, Nasen- und Ohrenkrankheiten, Kopf- und Halschirurgie

sowie

ARTORG Forschungszentrum für Biomedizinische Technik

Inhalt

1. Hintergrund 3
1.1 Anwendungen und Grenzen von Navigationsverfahren 3
1.2 Herausforderung Cochleaimplantation 4
2. Information zum Verfahren 5
2.1 Zusammenfassung bisher verfügbarer Literatur 5
2.2 Allgemeine Beschreibung des Systems 6
2.3 Funktionsprinzip 7
2.4 Benutzungsablauf 7
2.5 Erwartete Grenzparameter 8
2.6 Vorklinische Prüfung 8
2.7 Verfügbare klinische Daten 10
3. Studiendesign 10
3.1 Hypothese 10
3.2 Ziel der Studie 10
3.3 Endpunkte 10
3.4 Ablauf 11
3.5 Ein- und Ausschlusskriterien 12
4. Ethische Überlegungen / Risikomanagement 13
4.1 Identifizierung und Bewertung möglicher Risiken 13
4.2 Identifizierung und Bewertung von Nutzenaspekten 14
4.3 Zusammenfassung der Risiko-Nutzen-Abwägung 15
4.4 Unabhängige Datenüberwachung 16
4.5 Regeln für die Fortsetzung der Studie 16
4.6 Unterbrechung und Abbruch der Studie 16
5. Statistische Überlegungen 17
5.1 Studiendauer 17
6. Statistische Auswertung 17
7. Vorsichtsmassnahmen und Pflichten 17
7.1 Vorsichtsmassnahmen 17
7.2 Pflichten des Prüfers 18
8. Qualitätskontrolle und Qualitätssicherung 18
8.1 Gewährleistung des Zugangs 18
8.2 Umgang mit Daten und Proben 19

9. Andere Überlegungen 19
9.1 Kosten 19
9.2 Publikationen 19
10. Unterschriften 21

1. Hintergrund

1.1 Anwendungen und Grenzen von Navigationsverfahren

Stereotaktische Navigationsverfahren spielen in der Chirurgie seit über 10 Jahren eine bedeutende Rolle. Insbesondere in der Neuro-, HNO- und MKG-Chirurgie wird eine Vielzahl von Eingriffen nur noch unterstützt von Navigationssystemen durchgeführt. Hierbei orientiert sich der Chirurg an einem Bildschirm über die tatsächliche Lage der Instrumente im Körper des Patienten (Abbildung 1).

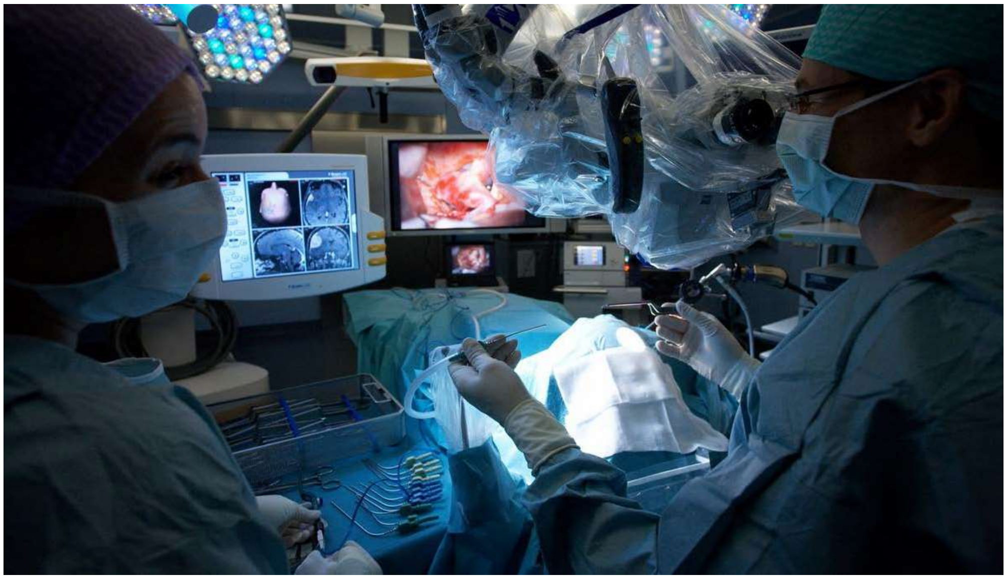

Abbildung 1: Auf einem Bildschirm wird die Lage von Instrumenten und relativ zu einem 3D Bilddatensatz des Patienten (typisch CT oder DVT) am Bildschirm eingeblendet. Der Chirurg kann Eingriffe durch eine verbesserte räumliche Orientierung präziser, schneller und weniger invasiv ausführen.

Die derzeit verfügbaren Verfahren und Ansätze für die stereotaktische Navigation sind aber für den mikrochirurgischen Einsatz ungeeignet, weil sie die erforderliche Gesamtgenauigkeit von < 0.5 mm [1], bei der resultierenden Instrumentenpositionierung nicht erreichen können. Navigationsverfahren können bisher nicht für die Implantation von Cochleaimplantation beziehungsweise für die Realisierung eines minimalinvasiven Zugangs durch den Mastoid herangezogen werden. Insbesondere bei der Instrumentenlokalisation im Mastoidbereich und zwischen den dort liegenden

Nerven (*Nervus Facialis* und *Chorda Tympani*) reicht die verfügbare Genauigkeit bisher nicht aus, um sicher zwischen diesen Strukturen navigieren zu können.

1.2 Herausforderung Cochleaimplantation

Die Cochleaimplantation ist eine etablierte Methode zur Rehabilitation von Gehörlosen oder hochgradig schwerhörigen Kindern oder Erwachsenen. Voraussetzung einer Cochleaimplantation ist die Herstellung eines Zugangs von der Aussenseite des Schädels in das Mittelohr um die Implantatelektrode in die Cochlea einbringen zu können.

In einer mehrstündigen Operation wird dazu der Mastoidknochen hinter dem Ohr (Grösse eines 2-Frankenstücks, siehe Abbildung 2) trichterförmig aufgebohrt (sogenannte Mastoidektomie). In der Tiefe des Mastoid werden durch den Operateur die anatomischen Landmarken und kritische Stellen wie Nervus Facialis und Chorda Tympani identifiziert. Zwischen diesen Nerven wird ein präziser Zugang zum Mittelohr realisiert (sogenannte posteriore Tympanotomie) und die Cochlea eröffnet. Danach kann die Elektrode in das Innenohr platziert werden. Das grossflächige Ausfräsen des Mastoids bei dieser konventionellen Operationsmethode ist ein zeitaufwendiger Prozess. Der resultierende Defekt stellt die grösste Invasivität der Operation dar und hat kosmetische und funktionelle Konsequenzen. Die Grösse der Mastoidektomie steht in keinem Verhältnis zur Grösse der einzubringenden Implantatelektrode.

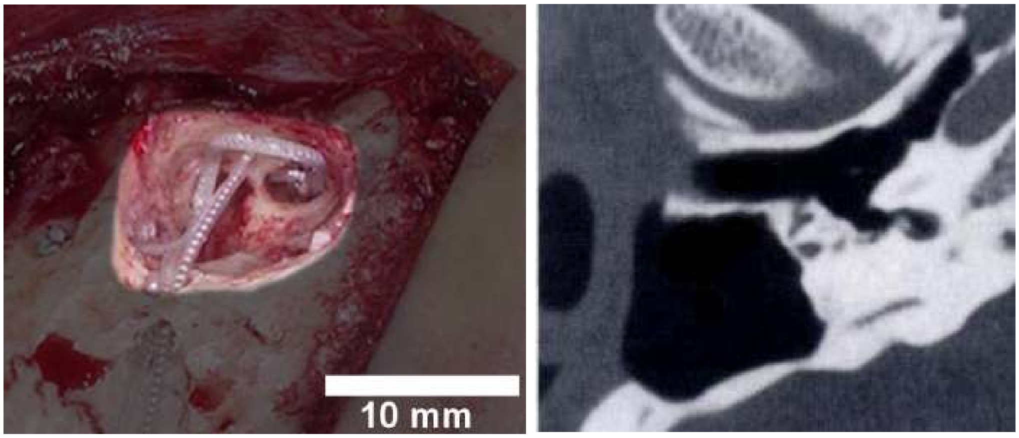

Abbildung 2: Konventionelle Mastoidektomie chirurgisch und radiologisch

Aus technischer Sicht wäre es ausreichend, die Mastoidektomie in Form einer kleinen Bohrung (Ø 1.5 mm) durchzuführen, in der die Implantatelektrode zwischen den Nerven und bis zum runden/ovalen Fenster in die Cochlea eingeführt werden könnte.

Dadurch könnte eine konventionelle Mastoidektomie vermieden werden. Dies würde sich in einer beträchtlichen Reduktion der Invasivität und damit verbundener Schmerzen auswirken. Eine dadurch mögliche leichtere Anästhesie und kürzere Operationsdauerwürde die Durchführung eines ambulanten Eingriffes ermöglichen (Abbildung 3).

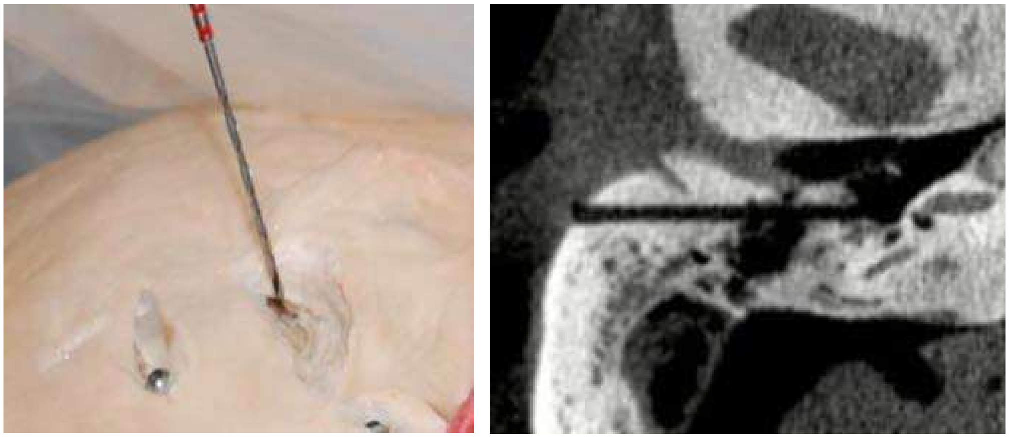

Abbildung 3: Minimalinvasiver Zugang zum Innenohr, chirurgisch und radiologisch

Unter HNO-Experten besteht Einigung darüber, dass ein derartiges minimalinvasives Vorgehen von hohem Interesse ist. Die geometrische Genauigkeit der bisher verfügbaren Navigationsansätze ist aber bisher nicht ausreichend, um eine sichere computerassistierte Bohrung im engen anatomischen Bereich zwischen *n. Facialis* und *Chorda Tympani* durchzuführen ohne diese wichtigen Strukturen zu schädigen.

2. Information zum Verfahren

2.1 Zusammenfassung bisher verfügbarer Literatur

In der Vergangenheit wurde bisher erfolglos versucht, mittels stereotaktischer computerbasierter Verfahren eine minimal-invasive Mastoidektomie mit anschliessender posteriorer Tympanotomie durchzuführen (Majdani et al. 2009, Klenzner et al. 2009, Baron et al. 2010 und Stieger et al. 2011). Hauptproblem war bisher die nicht erreichte Gesamtgenauigkeit der navigierten Verfahren.

Das sogenannte „Template-basierte Verfahren“ beruht auf der Herstellung von Bohrschablonen, die mittels einer Fräsmaschine und auf der Basis von geometrischen Informationen aus den patientenindividuellen CT-Daten hergestellt werden. Die Bohrschablone wird nach einer Sterilisierung an vorhandenen Knochenschrauben reproduzierbar und rigide befestigt. Über ein Führungsloch in der Schablone kann ein chirurgisches Bohrsystem eingeführt und exakt entlang einer vorher definierten Achse axial bewegt werden. Das Verfahren ermöglicht das Bohren eines Tunnelzugangs direkt in das Mittelohr. Bei Erreichen einer bestimmten vordefinierten Tiefe wird mittels intraoperativem DVT die korrekte Richtung (und damit die Sicherheit des Facialis) bestätigt (Labadie et al. 2010).

2.2 Allgemeine Beschreibung des Systems

Die Gesuchsteller arbeiten seit drei Jahren an der Entwicklung eines navigationsunterstützten Präzisionsansatzes um die minimalinvasive Implantation von Cochleaimplantaten zu ermöglichen.

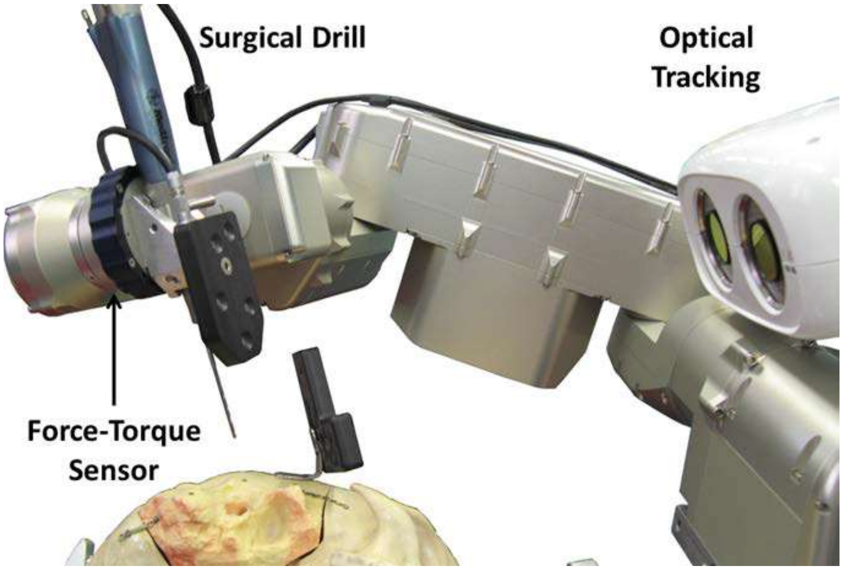

Abbildung 4: Komponenten des Navigationssystems

Während es weltweit bisher nicht gelungen ist, eine Gesamtgenauigkeit von unter 0.5 mm bei der Instrumentenführung zu erreichen, haben die Antragsteller diese Genauigkeitsanforderungen jetzt in verschiedenen Laborstudien und ebenfalls in einer Studie an Leichenschädeln mit 0.15 ± 0.08 mm (Bell et al 2013) klar erreicht. Daneben wurden in der Entwicklungsarbeit technische Aspekte zur Entwicklung eines dezidierten Instrumentenführungssystems [2], der Möglichkeit des Fräsens von Implantatta-

schen [2] sowie die Eignung für die Realisierung minimalinvasiver Zugänge zum Ohr [3] untersucht.

2.3 Funktionsprinzip

Der vorgestellte Ansatz beruht auf der Verwendung eines robotischen Instrumentenführungssystems in Kombination mit einem präzisionsoptischen Navigationssystem um präzise Fräsvorgänge am Mastoid zu ermöglichen. Das Verfahren verwendet Knochenschrauben als künstliche anatomische Landmarken, die bereits zu einem Zeitpunkt vor der 3D-Bildgebung implantiert werden. Die Landmarken werden in den Bilddaten identifiziert und vor dem eigentlichen Bohren als Referenzpunkte durch Navigationssystem eingemessen und die Positionen am Patienten dann mit den Schraubenpositionen in den Bilddaten referenziert. Das Instrumentenführungssystem führt dann die vorher definierten Bohr- und Fräsaufgaben interaktiv aus. Danach wird das Implantat konventionell implantiert, befestigt und aktiviert.

2.4 Benutzungsablauf

Ein minimal-invasives Vorgehen auf der Basis eines robotischen Führungssystems erfolgt auf der Basis eines validierten und exakt vorgeschriebenen Behandlungsablaufs (siehe Tabelle und Abschnitt Studienablauf).

Tabelle 1: Übersicht Benutzungsablauf

Sequenz	Beschreibung	Verantwortlich
**Vorbereitung**		
SEQ 0-1	Setzen von 5 Knochenschrauben im Mastoid	SURG
SEQ 0-2	Hochauflösendes DVT der Ohrregion	RAD
SEQ 0-3	Planung des Eingriffs am Computer	SURG
**Operation**		
SEQ 1-1	Einleitung der Anästhesie, Lagerung	ANÄS
SEQ 1-2	Hautschnitt zur Befestigung der optischen Referenz und für Zugang	SURG
SEQ 1-3	Aufbau und Vorbereitung Führungssystem	TECH
SEQ 1-4	Einmessen der Führungsschrauben	SURG
SEQ 1-5	Kontrolle der Genauigkeit	SURG

SEQ 1-6	Bohren des Tunnelzugangs	SURG
SEQ 1-7	Intraoperative Verlaufskontrolle mittels DVT	RAD
7	Entscheidung über Abbruch oder Fortsetzung des minimal-invasives Vorgehen	RAD / SURG
SEQ 1-8	Implantation der Elektrode und des Implantats	SURG
SEQ 1-9	Telemetrie	SURG
SEQ 1-11	Abschluss OP und Naht	SURG
**Nachbereitung**		
SEQ 2-1	Telemetrie Implantat	TECH
SEQ 2-2	Postoperative Bildgebung	TECH

2.5 Erwartete Grenzparameter

Tabelle 2: Erwartete Grenzparameter

Parameter	Beschreibung	Grenzwert
Effektive Bohrgenauigkeit	Geometrische Genauigkeit, mit der ein Tunnelzugang im Vergleich zur bildbasierten Planung effektiv gebohrt werden kann.	< 0.2 mm
Operationsdauer	Effektive Schnitt-Naht Zeit	60 min

2.6 Vorklinische Prüfung

2.6.1 Design

Ziel der vorklinischen Prüfung war der Nachweis der Einhaltung der Grenzparameter Genauigkeit und Operationsdauer anhand von humanen Kadaverpräparaten. Zusätzlich wurde in einer Tierstudie die Machbarkeit der Verwendung eines integrierten EMG Messsystems untersucht. Ausserdem wurde untersucht, inwieweit eine Implantatelektrode sicher durch ein Bohrloch implantiert werden kann.

2.6.2 In-vitro Tests

Erreichbare Gesamtgenauigkeit des Systems: In einer Studie an n=8 humanen Ganzkopfpräparaten wurde die erreichbare Gesamtgenauigkeit des Systems analysiert. Dabei wurden erreichbare Genauigkeiten bei der Instrumentenpositionierung von 0.08 ± 0.05 mm an der lateralen Schädelbasisoberfläche und 0.15 ± 0.08 mm an Positionen im Mittelohr nachgewiesen. Eine Beschreibung der Experimente befindet

sich im Anhang (im Anhang: Bell et al.: In Vitro Accuracy Evaluation of Image-Guided Robot System for Direct Cochlear Access, Otology & Neurotology 2013).

Machbarkeit EMG Integration: In einer Studie an n=5 Schafen wurde untersucht, inwieweit die Ableitung von EMG Signalen die Detektion des Facialis während des Bohrvorgangs ermöglichen würde. Es konnte gezeigt werden, dass eine sichere Detektion der Position des Nervs durch den Bohrer und während des Bohrvorgangs alleine nicht möglich ist. Trotzdem stellt das EMG-Mapping eine unverzichtbare Informationsquelle als qualitative Messmethode während einer Mastoidektomie und damit auch während des in dieser Studie geplanten minimalinvasiven Ansatzes dar. (im Anhang: Anso et al: Feasibility of Using EMG for Early Detection of the Facial Nerve During Robotic Direct Cochlear Access, Otology & Neurotology 2013)

Machbarkeit der Elektrodeninsertion: In einer Studie an n=8 humanen Ganzkopfpräparaten mit gebohrtem minimalinvasiven Zugang wurde die Machbarkeit einer Implantation (im Anhang: Wimmer et al. Cone Beam and Micro Computed Tomography Validation of Manual Array Insertion Following Minimally Invasive Robotic Direct Cochlear Access Audiology and Neurootology, in review).

2.6.3 Mechanische und elektrische Tests

Im Rahmen der elektrischen Prüfung des Gesamtsystems wurde eine Prüfung der elektromagnetischen Verträglichkeit des Systems (nach IEC 60601-1-2) durchgeführt. Das System ist unempfindlich gegen äussere Einflüsse und erzeugt keine gefährlichen EM-Emissionen. (siehe Anhang Testreport EM-Verträglichkeit). Eine Prüfung der elektrischen Sicherheit wurde ebenfalls erfolgreich durchgeführt und dokumentiert (siehe Anhang Testreport elektrische Sicherheit).

2.6.4 Zuverlässigkeitstests

Die Zuverlässigkeit des Systems in Hinblick auf die erreichbare Gesamtgenauigkeit wurde in einer Studie an humanen Ganzkopfpräparaten nachgewiesen.

2.6.5 Evaluation der biologischen Sicherheit

Die Anwendung des Systems ist aus biologischer Sicht sicher. Das Gesamtsystem kommt mit dem Patienten nur über das verwendete Bohrsystem in Berührung. Dieses ist medizinisch zugelassen.

2.7 Verfügbare klinische Daten

Im Bereich der bildgestützten und roboterassistierten minimalinvasiven Cochleaimplantation stehen bisher keine klinischen Daten zur Verfügung.

3. Studiendesign

3.1 Hypothese

Mit einem navigationsbasierten Instrumentenführungssystem können minimalinvasive Tunnelzugänge zum Mittelohr realisiert und Implantatelektroden korrekt implantiert werden.

3.2 Ziel der Studie

Nachweis der Machbarkeit eines kliniktauglichen Protokolls für das Fräsen von und das Implantieren mittels eines minimalinvasiven Zuganges zum Innenohr.

3.3 Endpunkte

3.3.1 Primäre Endpunkte

Gesamtgenauigkeit der erreichbaren Platzierung des Bohrloches gemessen als Target Registration Error (TRE) an der Aussenseite des Mastoid und im Mittelohr.

3.3.2 Sekundäre Endpunkte (technisch)

- Anzahl erfolgreich gefräster Tunnelzugänge
- Anzahl erfolgreich platzierter Implantatelektroden
- Genauigkeit der erreichbaren Registrierung über die geometrischen Abstände der Knochenschrauben in den Bilddaten (Fiducial Registration Error - FRE);
- Resultierende Abstände des Bohrloches zu allen beteiligten Strukturen;
- Zeitaufwände (Schnitt-Naht, sowie alle Teilschritte);

3.3.3 Sekundäre Endpunkte (klinisch)

- Unerwünschte Ereignisse: Verletzung n. Facialis oder Chorda Tympani
- Komplikationen: Temporäre Fazialisparese, Geschmacksstörungen, peri- und postoperative Blutungen, Infektionen

- Invasivität des Eingriffes: Konsum von Schmerzmittel postoperativ, Grösse der Wunde und Wundheilung, Inzisionen durch Schrauben und Bohrloch

3.4 Ablauf

3.4.1 Studieneinschluss

Auf Basis eines standardmässig vorliegenden diagnostischen CT/DVT wird entschieden ob der Patient minimal-invasiv operiert werden kann. Diese Entscheidung erfolgt auf Grund der anatomischen Grössen- und Platzverhältnisse im Innenohr (siehe Kapitel 3.5 Ein- und Ausschlusskriterien). Patienten, die sich für die Behandlungsform eignen, werden für die Studie angefragt. Die Anzahl der sich nicht für die minimal-invasive Behandlung eignenden Patienten wird erfasst.

3.4.2 Präoperative Vorbereitungen

1. **Implantation von Knochenschrauben:** Für die Einmessung des Systems werden vor der Operation vier Mini-Knochenschrauben (Medartis...) eingesetzt. Zusätzlich wird eine fünfte Verifikationsschraube platziert. Diese Knochenschraubenwerden in der Neurochirurgie ebenfalls für die Referenzierung standardmässig verwendet. Die Schrauben werden in den Mastoid und unter lokaler örtlicher Betäubung eingesetzt. Der entstehende Hautschnitt (ca. 2 mm) wird danach mit einem Pflaster abgedeckt.
2. **Präoperative DVT Bildgebung:** Von der Mastoidregion und Mittel- sowie Innenohr wird eine DVT-Aufnahme angefertigt.
3. **Computerbasierter Planung:** Mittels einer Computersoftware werden in den Bilddaten anatomische Strukturen wie n. Facialis, Chorda Tympani, Mastoid, Gehörknöchelchen, Rundes sowie ovales Fenster identifiziert, markiert und eine Bohrtrajektorie geplant. Die Planung wird als Computerdatei gespeichert und exportiert.

3.4.3 Intraoperativer Ablauf

1. Realisierung klinikähnlicher Bedingungen (OP-Tisch, Kopfklemme);
2. Lagerung des Patienten;
3. Anästhesie;
4. Montage & Inbetriebnahme des Instrumentenführungssystems;

5. Referenzierung über 4 Knochenschrauben;
6. Verifikation der verfügbaren Genauigkeit über die fünfte Schraube;
7. Bohren / Fräsen des DCA bis ca. 3 mm vor *n. Facialis*;
8. Anfertigen eines intraoperativen Kontroll-DVT
9. Entscheidung ob Bohrrichtung sicher ist
    a. **Ja:** Abschluss der Bohrung durch Instrumentenführung;
    b. **Nein:** Konvertierung zu konventioneller Mastoidektomie;
10. Implantation und Aktivierung des Implantats;

3.4.4 Post-operative Auswertung

1. Postoperative DVT-Bildgebung mit Segmentierung entsprechend Schritt 5;
2. Ko-Registrierung prä- und postoperative Bildgebung;
3. Vermessung des DCA in Bezug auf Planung, Vergleich mit präop. Planung;
4. Bestimmung Abstände DCA zu anatomischen Strukturen;
5. Auswertung

3.5 Ein- und Ausschlusskriterien

3.5.1 Einschlusskriterien

1. Unterschriebene Einwilligungserklärung
2. Schwangerschaftsausschluss
3. Alter > 18 Jahre
4. Indikation für Cochleaimplantation
5. Auf Basis eines aus der diagnostischen Abklärung vorliegenden CT/DVT Datensatzes wird anhand der Grösse des Facialis-Chorda-Abstands (>3 mm) geprüft, ob der Patient in die Studie eingeschlossen werden kann.

3.5.2 Ausschlusskriterien

1. Grösse Facialis-Chorda-Abstand <= 2.5 mm
2. Anatomische Malformationen im Mittel- oder Innenohr
3. Ungewöhnlicher Verlauf des Nervus facialis

4. Ethische Überlegungen / Risikomanagement

Als Antragsteller berücksichtigen wir eine Reihe von Risiken und Nutzenaspekten für eine ethische Güterabwägung.

4.1 Identifizierung und Bewertung möglicher Risiken

Dazu haben wir zunächst die zu erwartenden Eingriffsrisiken einer Bewertung hinsichtlich der Auftretenswahrscheinlichkeit und der zu erwartenden Konsequenzen bei Eintreten des Risikos unterzogen. Einerseits wird das Eintreten eines möglichen Risikos mit (unwahrscheinlich, entfernt denkbar, möglicherweise, sicheres eintreffen) bewertet. Anderseits werden die Auswirkungen auf die Operation (kein Einfluss, Veränderungen des Ablaufs, Abbruch), Belastungen für den Patienten (keine, geringe zusätzliche Belastung, grössere Belastungen) sowie Verletzungen des Patienten (kleinere Verletzung ohne Folgen, Verletzungen mit bleibenden Folgen, Tod) abgeschätzt und bewertet. In Tabelle 3 werden die identifizierten und bewerteten Risiken beschrieben.

Tabelle 3: Mögliche Nebenwirkungen und Risiken eines minimalinvasiven Eingriffs

Beschreibung des Risikos	Wahrscheinlichkeit des Auftretens	Konsequenz bei Eintreten
**R1: Zusätzliche Strahlenbelastung:** Bei allen Studienpatienten wird zusätzlich während und nach der Operation ein Computertomogramm des Ohres mittels eines speziellen digitalen Volumentomographen angefertigt. Pro Scan wird eine zusätzliche Strahlenbelastung von 0.1 mS[1] erwartet.	Sicher	Geringe zusätzliche Strahlenbelastung
**R2: Einbringen der Knochenschrauben:** Für die korrekte Referenzierung müssen insgesamt 5 Mini-Pins (ca. 2mm lang und 2 mm dick) in das Felsenbein eingeschraubt werden. Diese Pins werden in der Neurochirurgie standardmässig verwendet (CE zertifiziert). Die Schrauben werden nach der Operation entfernt.	Sicher	Minimale zusätzliche Invasivität
**R3: Persistierende Trommelfellperforation.** Während der Implantation eröffnet der Chirurg das Trommelfell zur visuellen Kontrolle durch einen kurzen ca. 4 mm langen Schnitt. Nach der Operation heilt das Trommelfell (TF) spontan, innerhalb von einigen Tagen bis Wochen wieder zu. In weniger als 1-2% der Fälle tritt eine länger andauernde Trommelfellperforation auf, die zusätzlich ambulant versorgt werden müsste.	unwahrscheinlich	Bei Persistenz, ambulanter Eingriff zur Schliessung der TF-Perforation
**R4: Temporäre Fazialisparese:** Durch die mechanische Bearbeitung des Mastoids kann der Nervus fazialis durch das Fräsen	unwahrscheinlich	Vorübergehende Parese des

[1] Kopfscan mittels eines digitalen Volumentomographen (Xoran XCat). Strahlenbelastung ist vergleichbar mit der Strahlenexposition durch die natürlichen Hintergrundstrahlung (2.4 mS per annum) die ein Mensch in einem Zeitraum von 2 Wochen erhält.

am Mastoid (Vibrationen, Hitze) irritiert werden. Derartige Paresen treten sehr selten und unabhängig vom chirurgischen Vorgehen auf. Zur Vermeidung werden die Bohrer / Fräser gekühlt. Tritt postoperativ eine temporäre Parese auf, wird in der Regel mit Kortikosteroiden behandelt und die Parese erholt sich komplett innerhalb von Tagen. Eine transitorische geringgradige Parese kann in seltenen Fällen länger dauern.		Gesichtsnerven.<br><br>Medikamentöse Behandlung mit Kortikosteroiden.
**R5: Gefahr einer Verletzung des n. Fazialis:** Eine Verletzung des N. fazialis ist das grösste anzunehmende unerwünschte Ereignis in dieser Studie. Derartige Verletzungen während klassischer Mastoidektomien treten nur extrem selten[2] (<<1%) und sind mit anatomischen Missbildungen oder Rezidiv-Operationen assoziiert. Zur Minimierung dieses Risikos während der Studie haben wir drei Sicherheitssysteme vorgesehen um die Integrität des Nervus Fazialis zu überwachen:<br>1. Kontinuierliches Fazialismonitoring (EMG) über Gesichtselektroden während des Bohrvorgangs<br>2. Intraoperative Bildgebung in der Mitte des Bohrvorgangs und in sicherer Entfernung zum N. Facialis (mittels DVT) zum Nachweis, dass die Bohrtrajektorie in sicherer Entfernung vom Facialis verläuft.<br>3. Verwendung eines oszillierenden Bohrsystems zur Vermeidung von Weichteilverletzungen.	unwahrscheinlich	Evtl. bleibende Parese
**R6: Gefahr einer Verletzung der Chorda Tympani: (**siehe auch R5): Eine Verletzung der Chorda Tympani kann zu transitorischen oder permanenten Geschmacksstörungen der betroffenen Zungenhälfte führen. In der Regel ist keine Einschränkung der Lebensqualität zu erwarten. Wir planen, die Chorda Tympani während des Eingriffs zu erhalten und während des Bohrvorganges entsprechend zu schützen (siehe Massnahmen in R5).	unwahrscheinlich	vorübergehende Beeinträchtigung des Geschmackssinns der betroffenen Zungenhälfte
**Wundinfektion/Wundheilungsstörung:** Es besteht das übliche Infektionsrisiko wie bei einem konventionellem Eingriff. Wundheilungsstörungen sind eher von der Konstitution des Patienten abhängig.	Gleiches Risiko wie bei konventioneller Chirurgie	Antibiotische Behandlung/offene Wunde

4.2 Identifizierung und Bewertung von Nutzenaspekten

Im Gegensatz dazu erwarten wir die in Tabelle 4 aufgeführten unmittelbaren Nutzen für die Studienteilnehmer:

Tabelle 4: Mögliche Nutzenaspekte bei einem minimalinvasiven Eingriff

[2] Nilssen EL, Wormald PJ.: Facial nerve palsy in mastoid surgery. J Laryngol Otol. 1997 Feb;111(2):113-6.

Beschreibung des Nutzenaspektes	Wahrscheinlichkeit des Auftretens
**Kürzer OP-Zeit:** Wir gehen davon aus, dass durch das minimalinvasive Vorgehen die OP-Zeit verkürzt wird.	Sehr wahrscheinlich
**Sanftere Anästhesie:** Wir gehen davon aus, dass die Schmerzmittelgabe während der Operation reduziert werden kann, da wesentlich weniger Knochenmaterial entfernt werden muss.	wahrscheinlich
**Verkürzung der Hospitalisierungsdauer:** Aufgrund eines schonenden Eingriffs und einer leichteren Anästhesie schätzen wir, dass die Patienten bereits nach 0 bis 1 Tag sicher entlassen werden können.	wahrscheinlich
**Kürzere Einheilungsdauer:** bedingt durch den weniger grossen Knochendefekt, den ein minimalinvasives Vorgehen hinterlässt.	Sehr wahrscheinlich
**Kosmetischer Effekt:** Wir gehen zusätzlich davon aus, dass ein minimalinvasives Vorgehen positive kosmetische Effekte (kleinere Narben) haben wird.	Sehr wahrscheinlich

4.3 Zusammenfassung der Risiko-Nutzen-Abwägung

Die Antragsteller haben eine Reihe von Risiken identifiziert und schätzen, dass eine Verletzung des N. Facialis das problematischste zu erwartende Ereignis in der Studie ist. Aus diesem Grunde wurden (schon lange vor Beginn dieser Studie) eine Reihe von Verfahren in das Studienvorgehen integriert, mit der zum Einen die Auftretenswahrscheinlichkeit einer Facialisverletzung reduziert (intraoperative Verlaufskontrolle, kontinuierliches EMG Monitoring während des Bohrens) und zum Anderen die Auswirkungen einer möglichen Berührung des Facialis durch den Bohrer (mittels Verwendung eines oszillierenden weichteilschonenden nicht schneidenden Bohrers) reduziert werden sollen. Die quantitative Bewertung des Restrisikos ist nicht abschliessend möglich, wir gehen aber davon aus, dass der Einsatz der genannten drei Sicherheitsmechanismen ein sicheres Vorgehen ermöglicht.

Weitere verfahrensbedingte Nebenwirkungen (Verwendung von Knochenschrauben, Anfertigen eines zusätzlichen intraoperativen DVT) werden im Vergleich zu den erwartbaren Vorteilen (OP-Zeit, Anästhesie, Heilungsprozess, Kosmetik) eines minimalinvasiven Vorgehens als vertretbar angesehen.

Fazit: Das von vielen Fachvertretern in der HNO-Chirurgen seit langer Zeit geforderte minimalinvasive Vorgehen kann erstmals im Rahmen einer klinischen Studie untersucht werden. Die dafür nach unserem Ermessen erforderlichen notwendigen Voraussetzungen hinsichtlich Präzision (Effizienz und Sicherheit) aber auch Sicherheit

wurden in aufwendigen Vorarbeiten durch die Antragsteller geleistet. Diese Studie wird bei Bewilligung die erste ihrer Art in der Welt sein.

4.4 Unabhängige Datenüberwachung

Da die geplante Studie die erste ihrer Art in der Welt sein wird und keinerlei anderen klinischen Daten zum geplanten Vorgehen vorliegen, sehen die Prüfer die Etablierung eines unabhängigen Panels bestehend aus folgenden drei internationalen Experten vor:

- Prof. Wolf-Dieter Baumgartner (Universitätskrankenhaus Wien, Österreich)
- Prof. Andreas Dietz (Universitätsklinikum Leipzig, Deutschland)
- Prof. D. Bodmer (Universitätsklinik Basel, Schweiz)

Ziel ist es, vorurteilsfreie und unbefangene Bewertung des Studienfortschritts zu erreichen. Die Mitglieder des Panels haben Zugang zu allen im Rahmen der Studie erhobenen Daten. Insbesondere entscheiden die Panelmitglieder nach jedem Eingriff über die Fortsetzung der Studie.

4.5 Regeln für die Fortsetzung der Studie

Anhand der studienspezifisch aufgezeichneten Daten (CRF, Bilddaten) entscheidet das unabhängige Panel nach jedem Eingriff über die Weiterführung der Studie und damit die Durchführung des nächsten Eingriffs. Die Entscheidung zur Fortsetzung der Studie muss einstimmig und schriftlich durch jedes Mitglied zu Händen des Studienleiters erfolgen. Die Entscheide werden mit den übrigen Studienunterlagen archiviert.

4.6 Unterbrechung und Abbruch der Studie

Sollte das unabhängige Panel nach Sichtung der Unterlagen zu der Einsicht gelangen, dass die Studie unterbrochen beziehungsweise abgebrochen werden soll, ist der Studienleiter zu informieren. Dieser wiederum informiert KEK Bern und Swissmedic. Über die Fortsetzung der Studie wird gegebenenfalls zu einem späteren Zeitpunkt entschieden.

5. Statistische Überlegungen

Die vorliegende Studie ist als Pilotstudie mit dem Ziel eines generellen Machbarkeitsnachweises des Verfahrens geplant. Dafür ist ein Patienteneinschluss von 3+10 Patienten vorgesehen. In einer Vorbereitungsphase möchten wir zunächst an bis zu drei Patienten evaluieren, ob alle bisherigen im Labor gemachten Annahmen hinsichtlich Benutzungsablauf (Schraubenpositionierung, Bildgebung) und klinischer Integration auch unter klinischen Bedingungen erfolgreich umgesetzt werden können. Dazu wird es unter Umständen nicht erforderlich sein, mit dem System das eigentliche minimal-invasive Vorgehen zu realisieren, sondern lediglich einzelne Teilschritte des Verfahrens zu überprüfen. Nach dieser Initialisierungsphase werden wir die eigentliche Pilot-Studie beginnen.

Wichtig: Durch die Studie können keine Fragen zur Sicherheit des Verfahrens beantwortet werden. Insbesondere zur Inzidenz von Facialis-Verletzungen werden wir in dieser Studie keine Aussagen treffen werden können.

5.1 Studiendauer

Wir gehen davon aus, dass wir bei einem (bisherigen) Patientenaufkommen von 0.5 Fällen pro Woche und einem Einschluss von mindestens 50% der erwachsenen Patienten mit einer effektiven Studiendauer von mindestens einem Jahr zu rechnen ist.

6. Statistische Auswertung

Alle erhobenen Daten werden statistisch deskriptiv ausgewertet.

7. Vorsichtsmassnahmen und Pflichten

7.1 Vorsichtsmassnahmen

Studienobjekt ist die Durchführung einer minimal-invasiven Mastoidektomie, die für die Implantation eines Cochleaimplantats erforderlich ist. Während der eigentliche Zugang zum Mittelohr mittels des oben genannten Führungssystems realisiert wird, sind alle anderen Bestandteile der medizinischen Behandlung sowohl vor (Bildgebung, OP-Vorbereitung, Anästhesie) als danach (Cochleostomie, Implantation, Implantatanpassung, OP-Abschluss) Routine. Als Vorsichtsmassnahmen wurden durch

die Antragsteller alle im Bereich der Risikominimierung genannten Verfahren genannt. Eine Kontrolle auf Schwangerschaft findet im Rahmen der sonstigen OP-Vorbereitung statt. Die postoperative Betreuung des Patienten erfolgt im Rahmen der festgelegten Richtlinien.

Für die Studie ist ausserdem die explizite Auswertung jedes einzelnen Eingriffes anhand der im Case Report Form erhobenen Daten vorgesehen. Das unabhängige Data-Monitoring Panel entscheidet nach jedem Eingriff eigenständig über die Fortsetzung der Studie. Sollte die Studie abgebrochen werden, wird die Ethikkommission sofort und innerhalb der vorgeschriebenen Fristen informiert.

7.2 Pflichten des Prüfers

7.2.1 Bestätigung

Mit ihrer Unterschrift bestätigen die Prüfer, dass die Studie gemäss Protokoll, GCP, und den geltenden gesetzlichen Bestimmungen durchgeführt wird.

7.2.2 Berichterstattung

Die Prüfer werden jegliche schwerwiegenden unerwünschten Ereignissen, Protokoll- sowie sonstige Änderungen in der Studie, sowie jegliche Zwischen- und Abschlussberichte der Swissmedic und der KEK Bern zur Verfügung stellen.

7.2.3 Stellungnahme zur Deckung von Schäden

Die Studie ist über die geltende Versicherung des Inselspitals versichert. Patienten werden im Rahmen der Studie kostenlos medizinisch betreut.

8. Qualitätskontrolle und Qualitätssicherung

8.1 Gewährleistung des Zugangs

Die Prüfer sichern der KEK Bern, der Swissmedic sowie den Mitgliedern des Datenpanels zu, jederzeit in die Originaldaten Einsicht zu nehmen, Audits durchzuführen, sowie Inspektionen durch Behörden und Durchführung von Monitoring Massnahmen zu ermöglichen.

8.2 Umgang mit Daten und Proben

8.2.1 Datenschutz

Alle im Rahmen der Studie erhobenen Daten werden anonymisiert erfasst. Alle Daten verbleiben in der Schweiz. Daten können in anonymisierter Form für Publikationen verwendet werden.

8.2.2 Archivierung

Alle im Rahmen der Studie erhobenen Daten werden für 5 Jahre archiviert. Davon unberührt sind die Archivierungspflichten im Rahmen der Implantation (15 Jahre) die aber nicht Bestandteil der Studie sind.

8.2.3 Vernichtung

Nach Ablauf der Aufbewahrungspflicht werden die Daten fachgerecht vernichtet.

9. Andere Überlegungen

9.1 Kosten

Kosten für die den eigentlichen Eingriff und das Implantat gehen zu Lasten der individuellen Krankenversicherung. Zusatzkosten für die Durchführung der Studie werden aus Forschungsmitteln finanziert. Siehe dazu auch das Dokument Budgetierungsplan.

9.2 Publikationen

Die Ergebnisse der Studie werden in geeigneten Publikationsorganen veröffentlicht.

11. Referenzen

1. J. Schipper, T. Klenzner, A. Aschendorff, I. Arapakis, G. Ridder and R. Laszig, "Navigation-controlled cochleostomy. Is an improvement in the quality of results for cochlear implant surgery possible?," HNO, vol. 52, no. 4, pp. 329-335, 2004.
2. Bell B., Gerber N., Salzmann J., Nielsen E., Zheng G., Stieger C., Nolte L.P., Caversaccio M., Weber S. (2010): Improving System Accuracy in Computer Aided Robotic ORL Surgery, in Conf Proc Hamlyn Symposium on Medical Robotics, GZ Yang and A Darzi (Eds.), 25 May, The Royal Society, London UK, pp11-12
3. Bell B., Roder S., Gerber N., Gavaghan K., Stieger S., Caversaccio M., Weber S. (2011): Computerassistierte Präzisionschirurgie am Ohr, AT-Automatisierungstechnik, accepted for publication

4. Bell B., Roder S., Gerber N., Gavaghan K., Stieger S., Caversaccio M., Weber S. (2011): Accuracy study of a Purpose Built Robot System for Minimally Invasive Cochlear Implantation, submitted to Acta ORL, in review
5. Vrionis FD, Foley KT, Robertson JH, Shea JJ 3rd. (1997a): Use of cranial surface anatomic fiducials for interactive image-guided navigation in the temporal bone: a cadaveric study. Neurosurgery. 1997 Apr;40(4):755-63;
6. Vrionis FD, Robertson JH, Foley KT, Gardner G. (1997b): Image-interactive orientation in the middle cranial fossa approach to the internal auditory canal: an experimental study. Comput Aided Surg. 1997;2(1):34-41.
7. A. Hussong, T. S. Rau, T. Ortmaier, B. Heimann, T. Lenarz, and O. Majdani, “An automated insertion tool for cochlear implants: another step towards atraumatic cochlear implant surgery,” International journal of computer assisted radiology and surgery, vol. 5, no. 2, pp. 163–171, Mar. 2010.
8. O. Majdani et al., “Force measurement of insertion of cochlear implant electrode arrays in vitro: comparison of surgeon to automated insertion tool,” Acta oto-laryngologica, vol. 130, no. 1, pp. 31–36, 2010.
9. J. Zhang, K. Xu, N. Simaan, and S. Manolidis, “A pilot study of robot-assisted cochlear implant surgery using steerable electrode arrays.,” Medical image computing and computer-assisted intervention : MICCAI 2006 International Conference on Medical Image Computing and Computer-Assisted Intervention, vol. 9, no. 1, pp. 33-40, Jan. 2006.
10. J. Zhang, W. Wei, J. Ding, J. T. Roland, S. Manolidis, and N. Simaan, “Inroads toward robot-assisted cochlear implant surgery using steerable electrode arrays.,” Otology & neurotology : official publication of the American Otological Society, American Neurotology Society [and] European Academy of Otology and Neurotology, vol. 31, no. 8, pp. 1199-206, Oct. 2010.
11. Labadie RF, Mitchell J, Balachandran R, Fitzpatrick JM. Customized, rapid-production microstereotactic table for surgical targeting: description of concept and in vitro validation. International journal of computer assisted radiology and surgery. 2009;4(3):273–280.
12. Majdani O, Rau TS, Baron S, et al. A robot-guided minimally invasive approach for cochlear implant surgery: preliminary results of a temporal bone study. International journal of computer assisted radiology and surgery. 2009;4(5):475–486.
13. Baron S, Eilers H, Munske B, et al. Percutaneous inner-ear access via an image-guided industrial robot system. Proceedings of the Institution of Mechanical Engineers, Part H: Journal of Engineering in Medicine. 2010;224(5):633–649.
14. Stieger C, Caversaccio M, Arnold a, et al. Development of an auditory implant manipulator for minimally invasive surgical insertion of implantable hearing devices. The Journal of laryngology and otology. 2011;125(1984):1–9.
15. Klenzner T, Ngan CCCC, Knapp FBFBB, et al. New strategies for high precision surgery of the temporal bone using a robotic approach for cochlear implantation. European Archives of Oto-Rhino-Laryngology. 2009;266(7):955–960.
16. Labadie RF, Mitchell J, Balachandran R, Fitzpatrick JM. Customized, rapid-production microstereotactic table for surgical targeting: description of concept and in vitro validation. International journal of computer assisted radiology and surgery. 2009;4(3):273–280.
17. Majdani O, Rau TS, Baron S, et al. A robot-guided minimally invasive approach for cochlear implant surgery: preliminary results of a temporal bone study. International journal of computer assisted radiology and surgery. 2009;4(5):475–486.
18. Baron S, Eilers H, Munske B, et al. Percutaneous inner-ear access via an image-guided industrial robot system. Proceedings of the Institution of Mechanical Engineers, Part H: Journal of Engineering in Medicine. 2010;224(5):633–649.
19. Stieger C, Caversaccio M, Arnold a, et al. Development of an auditory implant manipulator for minimally invasive surgical insertion of implantable hearing devices. The Journal of laryngology and otology. 2011;125(1984):1–9.
