# Supplementary material for: Robotic middle ear access for cochlear implantation: First in man
Source: PLoS One. 2019 Aug 2;14(8):e0220543. doi: 10.1371/journal.pone.0220543 (PMC6677292; doi:10.1371/journal.pone.0220543)
Supplement: S2 File — (PDF) [file pone.0220543.s002.pdf]

Clinical Trial Protocol

Proof-of-Concept Study

Robotic Cochlear Implantation

Translated Version 1.0

26th June 2016

Applicants

University Hospital for Ear, Nose, Throat, Head and Neck Surgery, Inselspital, Bern

and

ARTORG Centre for Biomedical Engineering

University of Bern, Switzerland

1. Background 3
1.1 Applications and limitations of surgical navigation systems 3
1.2 Challenges in cochlear implantation 4
2. Information on the procedure 5
2.1 The current state of the art 5
2.2 General information on the robotic CI system 6
2.3 Functional approach 7
2.4 Procedure 7
2.5 Expected edge/border parameters 8
2.6 Pre-clinical studies 8
2.7 Available clinical data 11
3. Clinical trial design 11
3.1 Hypothesis 11
3.2 Aim of the clinical trial 11
3.3 Study end points 11
3.4 Trial procedure 12
3.5 Inclusion- and exclusion criteria 13
4. Ethical considerations / Riskmanagement 14
4.1 Identification and assessment of potential risks 14
4.2 Benefits identification and assessment 15
4.3 Summary of the cost-benefit analysis 16
4.4 Independent data monitoring 17
4.5 Trial parameters to continue clinical study 17
4.6 Trial parameters to interrupt or abort clinical study 17
5. Statistical considerations 17
5.1 Duration of the clinical study 18
6. Statistical analysis 18
7. Precautions and responsibilities 18
7.1 Precautions 18
7.2 Assessors' obligations 19
8. Quality control and assurance 19
8.1 Access undertakings 19
8.2 Use of data and samples 19
9. AOB 20
9.1 Costs 20
9.2 Publication 20

10. Signatures 21

1. Background

1.1 Applications and limitations of surgical navigation systems

For the last 10 years stereotactic navigation systems have played a significant role in surgical interventions. During navigation-based surgery the clinician uses a screen to ascertain the orientation and position of the surgical instruments and anatomical structures in the patient. Particularly in neurological, ENT and maxillofacial surgery numerous procedures are principally carried out using navigation systems, (see Fig 1).

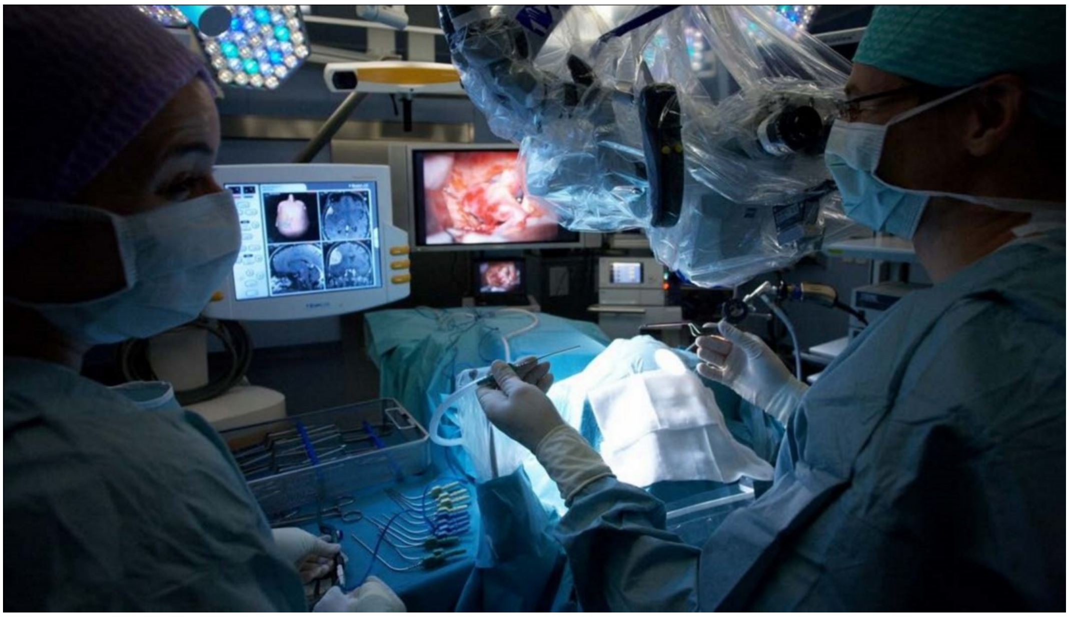

Fig 1: On a projection of patient CT 3D imaging data, surgical instruments are aligned and visualised. The improvement in spatial orientation enables the surgeon to carry out interventions with greater precision, reduced invasiveness and in many cases in less time.

Current state-of-the art in stereotactic navigation, despite its prevalence in other surgical interventions is unsuitable for microsurgical procedures, which require an overall precision of < 0.5 mm [1], including imaging of instrument alignment. For instance, existing navigation approaches do not permit minimally-invasive access to the cochlea through the mastoid bone to perform cochlear implantations (CIs). Damage to the major innervation of the mastoid, the *nervus facialis* (facial nerve) and the *chorda tympani*, must be avoided during CI surgery: When surgical tools are visualised at current levels of accuracy, safe navigation past these nerve structures is not possible using imaging alone.

1.2 Challenges in cochlear implantation

Cochlear implantation is part of standard clinical care to restore hearing in adults and children with severe to profound sensorineural hearing loss (SNHL). Cochlear implantation requires access from the side of the skull into the inner ear to insert the implant electrode into the cochlea.

The average surgical procedure for a CI takes several hours, sometimes less depending on the skill and experience of the surgeon. Following an incision behind the ear, the underlying skull bone, the mastoid, is opened by milling away the bone into a funnel that can be as large as the size of a £2/€2 coin (mastoidectomy, see Fig 2). The surgeon uses common, anatomical landmarks that are verified through visual inspection of the funnel-shaped opening, to identify the position of critical, neurological structures: the facial nerve and the *chorda tympani*. A posterior tympanotomy is the creation of middle ear access that passes between the facial nerve and the *chorda tympani*, followed by opening of the cochlea. Through this route the electrode of the CI is placed into the inner ear. The extensive milling away of mastoid bone during conventional CI is a time consuming stage in CI implantation. The opening created in the mastoid is considerably larger than the electrode array that is introduced through it into the inner ear. The large size of the access tunnel is currently unavoidable, as the surgeon uses visual cues and landmarks for orientation purposes, and these are only visible through a sufficiently large opening. By reducing the extent to which the mastoid bone needs to be removed, one aspect of invasiveness and possible functional and cosmetic sequelae could be improved.

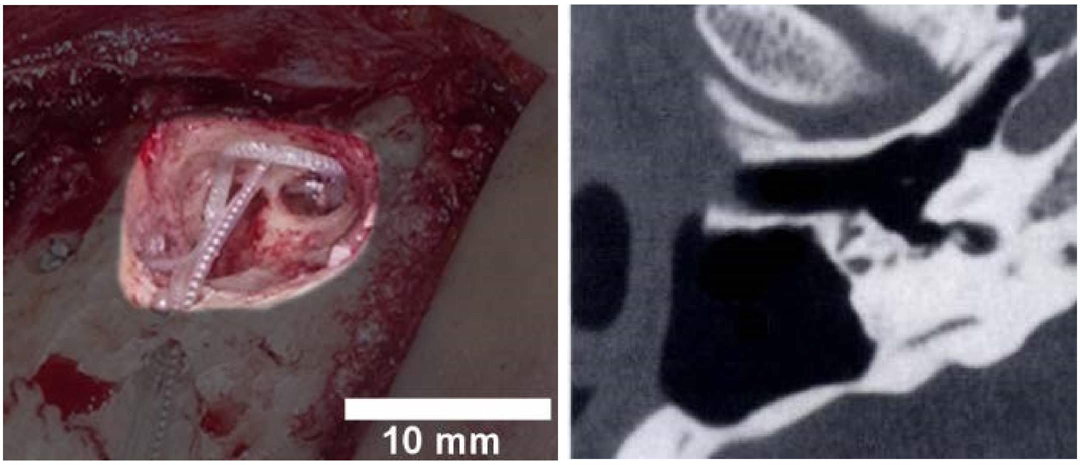

Fig 2: Conventional mastoidectomy surgical and radiological image

Considering the size of the electrode that needs to be manipulated and introduced into the cochlea, it could be argued that a smaller mastoidectomy with a bore size of (Ø 1.5 mm) would suffice to thread the electrode array past the nerves and into the round window opening of the cochlea. Shorter procedure times, a smaller bone lesion as part of an overall reduction in invasiveness and the potential for reduced anaesthesia could make outpatient/day-case/ambulant CI procedures more widely available (Fig 3).

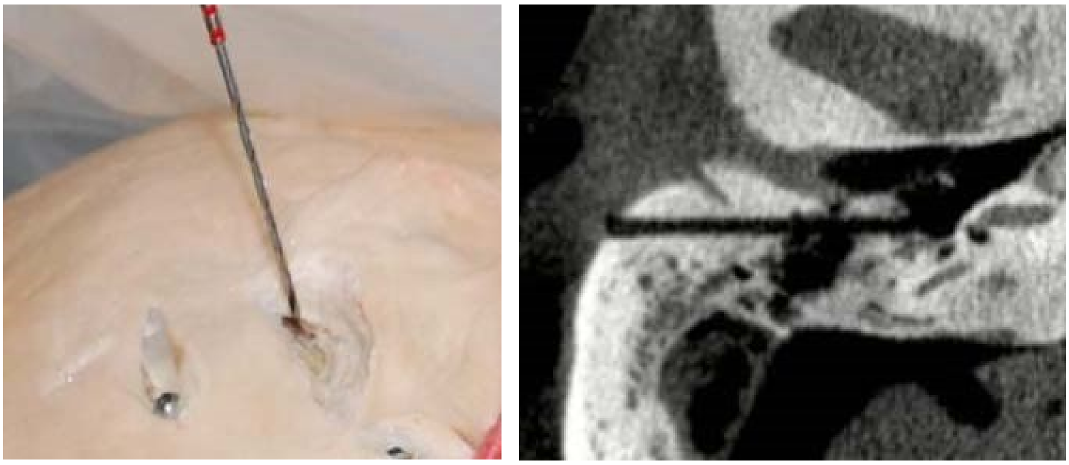

Fig 3: Minimally-invasive access to the inner ear surgical and radiological image

Key Opinion Leaders (KOLs) and stakeholders are in agreement, that innovations to enable navigation-led, high-precision CI are desirable and would lead to increased patient benefit. The end-to-end geometric accuracy of existing stereotactic navigation systems is insufficient to ensure safe drilling through the confined anatomy between the facial nerve and the *chorda tympani*, without damage to these vital structures.

2. Information on the procedure

2.1 The current state of the art

Past attempts to carry out computer navigation-assisted, micro-scale mastoidectomies and posterior tympanotomies were unsuccessful, as the mandatory and safe geometric accuracy could not be achieved. (Majdani et al. 2009, Klenzner et al. 2009, Baron et al. 2010 und Stieger et al. 2011).

Other approaches have included the use of stencil-like, drilling templates, which are based on patient-specific CT data. The plastic templates are fabricated, sterilised and secured via bone screws to the side of the patient's head. The template's guide holes can anchor a surgical drill platform to permit exact drill movement in the axial plane, directly into the middle ear. Correct depth and alignment to protect the facial nerve, are monitored using intra-operative Cone Beam CT, (Labadie et al. 2010).

2.2 General information on the robotic CI system

The applicants have concluded a six (6) year, multidisciplinary translation project to develop an image-based robotic surgery CI platform and surgical procedure. This novel approach will introduce the use of robotic technology for CI, with all associated advantages of maximal precision, standardisation and reduction in trauma.

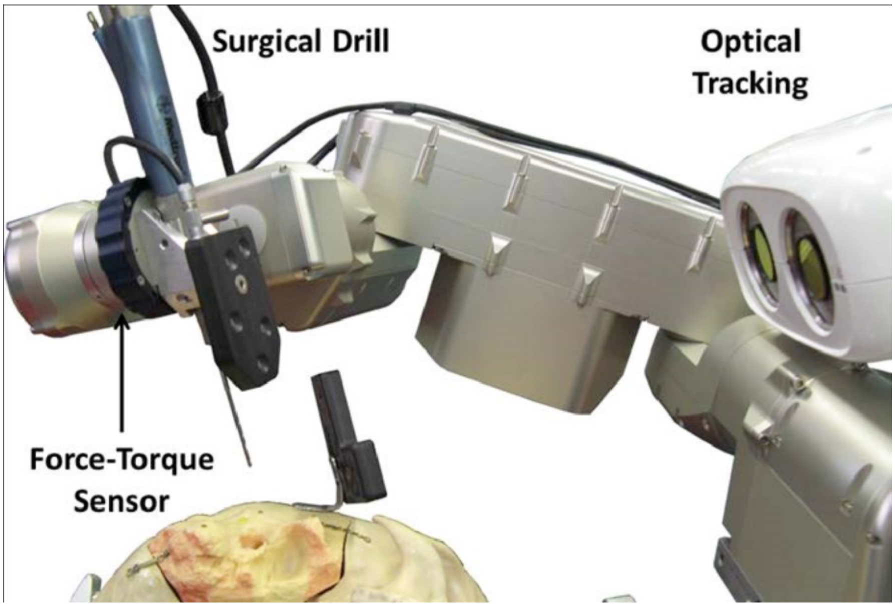

Fig 4: Components of the navigation and surgical platform

The navigation and surgical platform developed by the applicants can achieve overall accuracy for the handling of the surgical instruments of 0.15 ± 0.08 mm (Bell et al 2013), which was demonstrated in a number of laboratory studies and experiments on human cadaveric heads. The accuracy shown falls well within the safety margin of 0.5 mm required for safe drilling past the facial nerve and the *chorda tympani*. The dedicated system has been designed to perform high precision navigation of surgical instruments [2], the milling of implant beds and to permit minimally-invasive procedures in the inner ear [3].

2.3 Functional approach

The novel approach to CI proposed here combines a high-precision, robotic instrumentation-arm with an optical tracking system to carry out accurate drilling through the mastoid bone. The system employs bone screws implanted into the surface of the skull prior to pre-operative 3D imaging to serve as external landmarks. This involves tallying the position of the bone screws in the patients' head with the imaging data on the screws to act as references for the navigation of the robotic drilling arm. The surgeon is able to instruct the image-based guidance and robotic drill systems to carry out the drilling according to the pre-planned and safe pathway. Once the access is created, the implant is inserted, anchored and activated through the existing, standard manual procedure.

2.4 Procedure

A minimally-invasive CI procedure using a robotic navigation platform has to proceed along a validated and clearly defined treatment plan, (see Table 1 Study plan and 3.4 Trial procedure).

Table 1: Study plan

Step	Description	Responsible
**Pre-operative**		
SEQ 0-1	Insertion of 5 bone screws into the mastoid	SURG
SEQ 0-2	High-resolution CT scan of the inner ear	RAD
SEQ 0-3	Design and plan of drill trajectory using planning software (Otoplan™)	SURG
**Surgical procedure**		
SEQ 1-1	Anaesthetic and positioning of patient	ANAE
SEQ 1-2	Incision to attach optical tracker [patient tracker?] and to enable access to mastoid bone surface	SURG
SEQ 1-3	Set-up and prep of the optical tracking and robotic guidance system	TECH
SEQ 1-4	Calibration of bone screws	SURG
SEQ 1-5	Control of accuracy	SURG

SEQ 1-6	Drilling of access tunnel	SURG
SEQ 1-7	Intraoperative reference check using CBCT	RAD
7	Go/No-Go decision on minimally-invasive procedure or abort and completion using conventional CI procedure	RAD / SURG
SEQ 1-8	Electrode insertion and implantation of the CI	SURG
SEQ 1-9	Telemetry	SURG
SEQ 1-11	Surgery concluded and suture	SURG
**Post-operative**		
SEQ 2-1	Telemetry implant	TECH
SEQ 2-2	Postoperative CT imaging	TECH

2.5 Expected edge/border parameters

Table 2: Expected edge/border parameters

**Parameter**		**Cut-off**
End-to-end accuracy	Geometric accuracy of and deviation from actual drill trajectory when measured against CT image-based pre-planned route	< 0.2 mm
Duration of surgical procedure	Effective incision – suture time	60 min

2.6 Pre-clinical studies

2.6.1 Design

The aim of pre-clinical studies was to generate data confirming the accuracy and reproducibility with which the robotic platform is able to drill within the safe-zone into the mastoid and to determine the overall duration of a procedure using human cadaveric head specimens. In addition an *in vivo* proof-of-concept study was carried out in a sheep mastoid model to assess the utility of an integrated EMG stage during the drill procedure as an additional safety feature. The facility with which a CI electrode can be implanted through the minimally-invasive access route was also investigated in a human cadaveric study.

2.6.2 In-vitro assays

Overall accuracy of the system: A pre-clinical study on human cadaveric heads (n=8) determined overall accuracy of 0.08 ± 0.05 mm for instrument positioning and 0.15 ± 0.08 mm for several reference points in the middle ear. A summary of the study can be found in in the Appendix, Bell et al.: In Vitro Accuracy Evaluation of Image-Guided Robot System for Direct Cochlear Access, Otology & Neurotology 2013.

Proof EMG Integration: The use of EMG signals to sense and detect proximity to the facial nerve during the drilling procedure was investigated in a sheep mastoid model (n=5). The study did not conclusively show safe detection and position of the facial nerve via EMG. Nonetheless use of EMG-mapping represents important qualitative feedback during the mastoidectomy carried out as part of this minimally-invasive approach (see Appendix Anso et al: Feasibility of Using EMG for Early Detection of the Facial Nerve During Robotic Direct Cochlear Access, Otology & Neurotology 2013).

Proof-of-concept electrode insertion: A human cadaveric study (n=8) was carried out to assess the electrode array insertion and placement through the minimally-invasive access (see Appendix Wimmer et al. Cone Beam and Micro Computed Tomography Validation of Manual Array Insertion Following Minimally Invasive Robotic Direct Cochlear Access Audiology and Neurootology).

2.6.3 Mechanical and electrical tests

The electromagnetic compatibility of the entire system was carried out to meet the IEC 60601-1-2 standard. The system is resistant to external interference and does not emit EM radiation exceeding the limits set by the standard (see Appendix certification EM-compatibility). The system was also passed for electrical safety (see Appendix certification electrical safety).

2.6.4 Reproducibility

The reproducibility of the end-to-end accuracy was determined in a human cadaveric study.

2.6.5 Evaluation of biological safety

Use of the system on patients is considered generally safe. The only invasive contact of the system with the patients is through the drill-bit, which has licensed approval as a medical device.

2.7 Available clinical data

At present there are no clinical data available on the use of imaging-based, robotic CI using minimally-invasive access to the cochlea.

3. Clinical trial design

3.1 Hypothesis

Use of an imaging-based navigation system and robotic drilling platform to create minimally-invasive access to the middle ear and enable accurate placement of CI electrodes into the cochlea.

3.2 Aim of the clinical trial

Proof-of-concept of a clinically applicable surgical method for the milling and implantation of CIs through minimally-invasive access to the inner ear.

3.3 Study end points

3.3.1 Primary end points

Overall accuracy of drill-hole placement measured as Target Registration Error (TRE) at the outside of the mastoid and the middle ear.

3.3.2 Secondary end points (technical)

- Number of successfully drilled/milled access tunnels;
- Number of successfully placed electrode arrays;
- Accuracy of the registration obtainable from registration of the geometric positioning of the bone screws in the imaging data (Fiducial Registration Error - FRE);
- Distance between the drill-hole and all other relevant anatomical structures;
- Time-lines and duration (Incision – Suture, all other component stages);

3.3.3 Secondary end points (clinical)

- Adverse events: Injury or damage to the facial nerve or the *chorda tympani*
- Complications: Temporary facial paralysis, disruption of taste sensation, peri- and post-operative bleeding, infections;

- Invasiveness of the procedure: Post-operative use of pain relief, size of wound and speed of wound healing, size of incision for drill-hole and bone screws;

3.4 Trial procedure

3.4.1 Study participation

The choice of patient for a minimally-invasive CI procedure is made based on the diagnostic CT imaging as carried out in the course of standard clinical care for conventional CIs. The decision making parameters include the anatomical size and space present in the patient inner ear (see 3.5 Inclusion- and Exclusion criteria). Suitable patients are informed, invited and consented to participate in the study. In addition, unsuitable patients and their data are also logged

3.4.2 Pre-operative procedures

1. **Insertion of bone screws:** System calibration involves the use of five (5) mini bone screws, one of which is for reference purposes. The bone screws (e.g. Medartis brand) are used routinely in neurosurgical procedures. The insertion of the bone screws is done under local anaesthetic and the incision (2mm) and screw are covered with a plaster until the CI procedure, usually the next day.
2. **Pre-operative CT Imaging:** Patient CT images of the mastoid bone, the middle and inner ear regions are taken. High resolution temporal bone protocol?.
3. **Computer- assisted CI planning procedure:** This procedure is one of the three core steps of the novel minimally-invasive CI procedure in this study. Using the software platform Otoplan™ anatomical structures including: the facial nerve, *chorda tympani*, mastoid, the ossicles, and the round and oval window openings of the cochlea are identified, marked and used as landmarks to plan a safe drill trajectory. The plan is saved and exported in a computer file format.

3.4.3 Intraoperative procedures

1. Simulated Operating Theatre (OT) set-up: OT table, head-clamp;
2. Patient positioning;
3. Anaesthesia;
4. Assembly and navigation platform start-up;
5. Registration using four (4) bone screws;

6. Verification of accuracy with reference to the fifth (5) bone screw;
7. Drilling/Milling of the cochlear access route up to 3mm ahead of the facial nerve;
8. Intraoperative CBT imaging for verification of facial nerve position;
9. Go/No-go on drill trajectory
   a. **Yes:** Complete drilling using surgical navigation and robotic platform;
   b. **No:** Proceed by switching to conventional mastoidectomy;
10. Implantation and activation of the implant;

3.4.4 Post-operative analysis

1. Postoperative CT Imaging and segmentation according to step 5 in 3.4.2;
2. Co-registration of pre- and post-operative images;
3. Measurement of cochlear access relative to route set-out using pre-operative planning software (Otoplan™);
4. Confirmation of distance between minimally-invasive access route and anatomical structures;
5. Analysis

3.5 Inclusion- and exclusion criteria

3.5.1 Inclusion criteria

1. Signed consent form (see Appendix for draft)
2. Exclusion of pregnancy
3. Age > 18 years
4. Indicated for cochlear implantation
5. Based on the diagnostic CT Image generated as part of routine clinical care the space between facial nerve and *chorda tympani* is verified to be (>3 mm) to permit inclusion in the clinical trial.

3.5.2 Exclusion criteria

1. Space between facial nerve and *chorda tympani* less than <= 2.5 mm
2. Anatomical irregularities of the middle and inner ear
3. Unusual orientation and path of the facial nerve

4. Ethical considerations / Riskmanagement

The applicants will employ a cost-benefit analysis of the novel procedure to support the considerations for ethical approval of this study.

4.1 Identification and assessment of potential risks

The applicants have carried out a risk assessment of the probability of certain risk events occurring and any expected consequences in case an assessed risk event should occur. The chance of a risk event occurring is determined as a probability according to likelihood: unlikely, likely, highly likely and certain. In addition the consequences of a risk event occurring during the surgical procedure are also determined and assessed. They include: no effect, amendment of the procedure, abort procedure. Furthermore any additional burden on the patient in case of a risk event taking place are set-out. These include: none, minor additional burden, increased additional burden. Finally risk of injury and death of a patient are assessed to be: minor injuries with no permanent effects, injuries with permanent effects and death. Table 3 below summarises the risk-assessment in all the above areas of the novel procedure.

Table 3: Possible side-effects and risks of the minimally-invasive CI procedure

Summary of risk event	Likelihood of risk event	Consequences of risk event
**R1: Additional radiation exposure:** All trial participants will have to undergo two additional imaging procedures of the ear with a specialised scanner for volume computed tomography. Each scan will mean an additional radiation dose of 0.1 mS[1].	Certain	Minor, additional radiation burden
**R2: Attachment of the bone screws:** For correct registration a total of 5 mini-pins (approx. 2 mm long and 2 mm thick) have to be inserted into the temporal bone. The bone screws are used in routine clinical care in neurosurgery (they are CE marked). The bone screws are removed at the end of the CI surgery.	Certain	Minimal, additional invasiveness
**R3: Persistent [“Permanent“] perforation of the ear drum.** During implantation the ear drum is opened about 4 mm to allow visual inspection. Following the procedure, the ear drum heals [spontaneously] over a period of a few days or weeks. In around 1-2% of all cases the ear drum does not sufficiently heal and requires further treatment as an outpatient.	Unlikely	If incision does not heal, any ear drum perforation is surgically treated as an outpatient procedure
**R4: Temporary facial paralysis:** The mechanical agitation (vibration, heat) of the drilling through the mastoid can irritate the		Temporary paralysis of the

[1] Taking patient images using a scanner for volume computed tomography (Xoran® xCAT®) is comparable to the total natural background radiation experienced (2.4 mS per annum) by humans over a period of 2 weeks.

facial nerve, leading to temporary paralysis. This type of paralysis of the facial nerve is very rare and usually unrelated to the surgeon's approach to the procedure. To mitigate against this risk, the drill and the burr are water-cooled. In case of post-operative, temporary paralysis, treatment with corticosteroids completely resolves the paralysis within a few days. A transitory, lower-grade paralysis can occasionally persist for longer.	Unlikely	facial nerves. First-line therapy with systemic corticosteroids
**R5: Risk of injury to the facial nerve:** injury to the facial nerve is the most serious adverse event in this study. In conventional mastoidectomies, this is an extremely rare event[2] (<<1%) and is usually associated with anatomical anomalies or revision surgery. Three separate safety systems are used to monitor integrity of the facial nerve during the drilling phase:<br>1. Continuous EMG monitoring of the facial nerve via electrodes placed on the face of the patient<br>2. Intraoperative imaging at the half-way mark of the drill trajectory and at a safe distance from the facial nerve. This is to ensure that the drill path is within the safe-zone, away from the facial nerve<br>3. Use of an oscillating drill system to avoid soft-tissue injury.	Unlikely	Possible, permanent paralysis of the facial nerve
**R6: Risk of injury to the *chorda tympani*:** (see R5): Injury of the *chorda tympani* can lead to temporary or permanent impairment of the sense of taste on the same side of the tongue as the injury. In general this has no impact on the patient's quality of life. Nonetheless, the approach proposed here will seek to preserve the *chorda tympani* and protect its integrity during the drilling. (see measures in R5).	Unlikely	Temporary or permanent impairment of a sense the taste on the same side of the tongue as the injury
**Infection of the wound/Impaired wound healing:** The risk of a post-operative wound infection is comparable to that of conventional CI. Impaired wound-healing is dependent on the overall health and fitness of the patient	Same risk as that of conventional CI surgery	Antibiotic treatment/open wound

4.2 Benefits identification and assessment

Expected benefits to participants of the study are summarised in Table 4:

Table 4: Potential benefits of a minimally-invasive CI procedure

Benefit of minimally-invasive procedure	Likelihood
**Shorter procedure time:** The minimally-invasive procedure is expected to lead to a shorter CI procedure time.	Highly likely
**Lower anaesthetic dose:** It is expected that the level of pain relief required during and after the procedure will be reduced, as significantly less bone has to be removed.	Likely

[2] Nilssen EL, Wormald PJ.: Facial nerve palsy in mastoid surgery. J Laryngol Otol. 1997 Feb;111(2):113-6.

**Shorter hospital stay:** The gentler procedure and the reduced anaesthetic protocol could lead to discharge of patients as outpatients on the same day or one day post-procedure.	Likely
**Shorter healing time:** due to the reduced bone lesion, the overall healing time is likely to be reduced.	Highly likely
**Cosmetic benefits:** the minimally-invasive procedure is likely to have aesthetic advantages of smaller scars and less scar tissue	Highly likely

4.3 Summary of the cost-benefit analysis

The applicants have assessed a number of risks and consider injury to the facial nerve the most serious adverse event expected to occur during the procedure. The design of the trial includes a number of safety features that mitigate against this risk:

- Reducing the **probability of damage** to the facial nerve through intraoperative process control and continuous EMG monitoring during the drilling;
- Reducing **injury sequelae** of possible contact of the drill bit with the facial nerve by using an oscillating, soft-tissue sparing and non-lacerating drill.

Considering these risk reducing measures the remaining risk, although not easily quantified, is likely to be low and use of the three safety mechanisms will ensure patient safety during the procedure.

All other adverse events, including those associated with the use of bone screws, additional intraoperative imaging (radiation dose), are seen as acceptable when balanced against the benefits of shorter procedure time, lighter anaesthetic load, faster healing and aesthetic considerations.

In conclusion: There is widespread agreement among ENT Otology KOLs, that a minimally-invasive procedure for CI is timely. The applicants are proposing the first clinical trial of a system with the required precision, multiple redundant safety features and improved efficiency over the conventional CI procedure. Data from extensive pre-clinical investigations ensure that any remaining risks of a clinical study using this novel system is low and that patient safety is upheld. If approved, this clinical study will be the first of its kind worldwide.

4.4 Independent data monitoring

In the absence of any relevant clinical reference data for this completely novel procedure, the assessors would like to propose the establishment of an Independent, expert Advisory Panel (IAP) made up of three International ENT Otology KOLs: :

Independent, expert Advisory Panel- IAP

- Prof. Wolf-Dieter Baumgartner (Universitätskrankenhaus Vienna, Austria)
- Prof. Andreas Dietz (Universitätsklinikum Leipzig, Germany)
- Prof. D. Bodmer (Universitätsklinik Basel, Switzerland)

The role of the IAP is to ensure unencumbered and impartial assessment of progress in the study. The members of the IAP will have access to all data generated as part of the study and will approve the continuation of the study following each procedure.

4.5 Trial parameters to continue clinical study

Based on the data collected as part of each trial procedure (Case Report Form (CRF) and imaging data) the IAP will decide to continue the clinical study and approve the procedure on the next patient. The IAP decision has to be unanimous and delivered in writing to the Clinical Trial Lead (CTL). The IAP decisions are archived as part of the trial documentation.

4.6 Trial parameters to interrupt or abort clinical study

An IAP ruling on the interruption or aborting the clinical study following the analysis of data from a procedure must be communicated immediately to the CTL. The CTL must inform the KEK Bern and Swissmedic of the IAP's decision. Any possible continuation of the clinical trial will be considered at a later date.

5. Statistical considerations

The clinical trial proposed by the applicants is a general proof-of-concept pilot study of the novel procedure. A statistically significant number of participants would include 3 + 10 patients. The first stage of the pilot trial will involve confirming pre-clinical findings of various stages of the procedure (positioning of bone screws, imaging) on up to three patients, without carrying out the entire minimally-invasive intervention. Other initial

aspects will involve assessing integration into the clinical setting and surgical workflow prior to attempting a full, minimally-invasive procedure on patient 4.

Please note: This study is not designed to determine the safety of the procedure, or generate statistically significant data on the incidence of facial nerve injury.

5.1 Duration of the clinical study

The expected duration of the study is twelve (12) months. This is based on an average of 0.5 CI patients per week at the clinical centre and inclusion of 50% of all adult patients meeting inclusion criteria and consenting to the procedure.

6. Statistical analysis

Clinical data will be subject to descriptive, statistical analysis.

7. Precautions and responsibilities

7.1 Precautions

The clinical study specifically involves a minimally-invasive mastoidectomy, which is the route to implant a CI. Access to the middle ear will be created using the novel image-based navigation and robotic drilling platform. All other steps of the CI procedure in the study will be identical to conventional CI surgery. They will involve routine clinical intervention including: imaging, pre-operative preparation, general anaesthetic and during the procedure cochleostomy, electrode insertion, implant adjustment and completion of the surgery. Risk reduction processes for the minimally-invasive procedure in this clinical study are set-out above. Pregnancy is excluded as part of routine clinical care for surgical interventions. Post-operative care will follow the standard clinical care pathway for CI patients in Switzerland.

The clinical study will require detailed analysis and documentation of each patient intervention in a CRF. The IAP will collectively decide the continuation of the study following each intervention, and this is only permitted if the IAP decision is unanimous. In case the IAP decides to abort the study, the ethics committee must be informed within the official deadlines.

7.2 Assessors' obligations

7.2.1 Confirmation

The undersigned confirm that the study protocol conforms to Good Clinical Practice (GCP) and complies with all existing statutory requirements.

7.2.2 Monitoring

The assessors will ensure that all and any serious adverse events, changes to the study protocol and interim and final reports are made accessible to Swissmedic and the KEK Bern.

7.2.3 Statement of liability

The study is underwritten by the existing insurance provision of the Inselspital. All clinical costs are covered and patients will receive their treatment free-of-charge as part of the study.

8. Quality control and assurance

8.1 Access undertakings

The assessors undertake to ensure access to all primary data, to enable the IAP, KEK Bern, Swissmedic and any other official body to carry out inspections, audits and monitoring of the clinical study at any time.

8.2 Use of data and samples

8.2.1 Data protection

All data generated in this study will be routinely anonymised, and will remain archived in Switzerland, see also 8.2.2. Any data used in publication or dissemination activities under 9.2 will only be used in anonymised form.

8.2.2 Archiving

All data generated in this study will be archived for five (5) years. All data generated as part of routine clinical care for CI will be archived for fifteen (15) years; obligations on the latter data to not extend to this study.

8.2.3 Secure destruction of data

At the end of the archiving period, the study data records will be securely and verifiably destroyed.

9. AOB

9.1 Costs

The costs of the routine CI procedure and the implants will be covered by the health insurer. Additional treatment costs incurred as part of the clinical study will be covered from research funding (see Annex Clinical Study Budget).

9.2 Publication

The results of the study will be disseminated through relevant publications in the scientific and clinical literature and at conferences. The publications will be subject to all applicable data protection restrictions, see also 8.2.1.

11. References

1. J. Schipper, T. Klenzner, A. Aschendorff, I. Arapakis, G. Ridder and R. Laszig, "Navigationcontrolled cochleostomy. Is an improvement in the quality of results for cochlear implant surgery possible?," HNO, vol. 52, no. 4, pp. 329-335, 2004.
2. Bell B., Gerber N., Salzmann J., Nielsen E., Zheng G., Stieger C., Nolte L.P., Caversaccio M., Weber S. (2010): Improving System Accuracy in Computer Aided Robotic ORL Surgery, in Conf Proc Hamlyn Symposium on Medical Robotics, GZ Yang and A Darzi (Eds.), 25 May, The Royal Society, London UK, pp11-12
3. Bell B., Roder S., Gerber N., Gavaghan K., Stieger S., Caversaccio M., Weber S. (2011): Computerassistierte Präzisionschirurgie am Ohr, AT-Automatisierungstechnik, accepted for publication
4. Bell B., Roder S., Gerber N., Gavaghan K., Stieger S., Caversaccio M., Weber S. (2011): Accuracy study of a Purpose Built Robot System for Minimally Invasive Cochlear Implantation, submitted to Acta ORL, in review
5. Vrionis FD, Foley KT, Robertson JH, Shea JJ 3rd. (1997a): Use of cranial surface anatomic fiducials for interactive image-guided navigation in the temporal bone: a cadaveric study. Neurosurgery. 1997 Apr;40(4):755-63;
6. Vrionis FD, Robertson JH, Foley KT, Gardner G. (1997b): Image-interactive orientation in the middle cranial fossa approach to the internal auditory canal: an experimental study. Comput Aided Surg. 1997;2(1):34-41.
7. A. Hussong, T. S. Rau, T. Ortmaier, B. Heimann, T. Lenarz, and O. Majdani, “An automated insertion tool for cochlear implants: another step towards atraumatic cochlear implant surgery,” International journal of computer assisted radiology and surgery, vol. 5, no. 2, pp. 163–171, Mar. 2010.
8. O. Majdani et al., “Force measurement of insertion of cochlear implant electrode arrays in vitro: comparison of surgeon to automated insertion tool,” Acta oto-laryngologica, vol. 130, no. 1, pp. 31–36, 2010.

9. J. Zhang, K. Xu, N. Simaan, and S. Manolidis, “A pilot study of robot-assisted cochlear implant surgery using steerable electrode arrays.,” Medical image computing and computer-assisted intervention : MICCAI 2006 International Conference on Medical Image Computing and ComputerAssisted Intervention, vol. 9, no. 1, pp. 33-40, Jan. 2006.
10. J. Zhang, W. Wei, J. Ding, J. T. Roland, S. Manolidis, and N. Simaan, “Inroads toward robot-assisted cochlear implant surgery using steerable electrode arrays.,” Otology & neurotology : official publication of the American Otological Society, American Neurotology Society [and] European Academy of Otology and Neurotology, vol. 31, no. 8, pp. 1199-206, Oct. 2010.
11. Labadie RF, Mitchell J, Balachandran R, Fitzpatrick JM. Customized, rapid-production microstereotactic table for surgical targeting: description of concept and in vitro validation. International journal of computer assisted radiology and surgery. 2009;4(3):273–280.
12. Majdani O, Rau TS, Baron S, et al. A robot-guided minimally invasive approach for cochlear implant surgery: preliminary results of a temporal bone study. International journal of computer assisted radiology and surgery. 2009;4(5):475–486.
13. Baron S, Eilers H, Munske B, et al. Percutaneous inner-ear access via an image-guided industrial robot system. Proceedings of the Institution of Mechanical Engineers, Part H: Journal of Engineering in Medicine. 2010;224(5):633–649.
14. Stieger C, Caversaccio M, Arnold a, et al. Development of an auditory implant manipulator for minimally invasive surgical insertion of implantable hearing devices. The Journal of laryngology and otology. 2011;125(1984):1–9.
15. Klenzner T, Ngan CCCC, Knapp FBFBB, et al. New strategies for high precision surgery of the temporal bone using a robotic approach for cochlear implantation. European Archives of OtoRhino-Laryngology. 2009;266(7):955–960.
16. Labadie RF, Mitchell J, Balachandran R, Fitzpatrick JM. Customized, rapid-production microstereotactic table for surgical targeting: description of concept and in vitro validation. International journal of computer assisted radiology and surgery. 2009;4(3):273–280.
17. Majdani O, Rau TS, Baron S, et al. A robot-guided minimally invasive approach for cochlear implant surgery: preliminary results of a temporal bone study. International journal of computer assisted radiology and surgery. 2009;4(5):475–486.
18. Baron S, Eilers H, Munske B, et al. Percutaneous inner-ear access via an image-guided industrial robot system. Proceedings of the Institution of Mechanical Engineers, Part H: Journal of Engineering in Medicine. 2010;224(5):633–649.
19. Stieger C, Caversaccio M, Arnold a, et al. Development of an auditory implant manipulator for minimally invasive surgical insertion of implantable hearing devices. The Journal of laryngology and otology. 2011;125(1984):1–9.

Signature page removed.
